# MicroRNA-122 Regulation of HCV Infections: Insights from Studies of miR-122-Independent Replication

**DOI:** 10.3390/pathogens11091005

**Published:** 2022-09-02

**Authors:** Mamata Panigrahi, Michael A. Palmer, Joyce A. Wilson

**Affiliations:** Department of Biochemistry, Microbiology, and Immunology, University of Saskatchewan, Saskatoon, SK S7N 5E5, Canada

**Keywords:** Hepatitis C virus, miR-122, miR-122-independent replication, 5′ untranslated region, viral tropism

## Abstract

Despite the advancement in antiviral therapy, Hepatitis C remains a global health challenge and one of the leading causes of hepatitis related deaths worldwide. Hepatitis C virus, the causative agent, is a positive strand RNA virus that requires a liver specific microRNA called miR-122 for its replication. Unconventional to the canonical role of miRNAs in translation suppression by binding to 3′Untranslated Region (UTR) of messenger RNAs, miR-122 binds to two sites on the 5′UTR of viral genome and promotes viral propagation. In this review, we describe the unique relationship between the liver specific microRNA and HCV, the current knowledge on the mechanisms by which the virus uses miR-122 to promote the virus life cycle, and how miR-122 impacts viral tropism and pathogenesis. We will also discuss the use of anti-miR-122 therapy and its impact on viral evolution of miR-122-independent replication. This review further provides insight into how viruses manipulate host factors at the initial stage of infection to establish a successful infection.

## 1. Introduction

Hepatitis C Virus (HCV) is a blood borne pathogen and one of the leading causes of chronic liver disease worldwide. It has a global prevalence of 1%, corresponding to 71.1 million infections [1], and according to a report by World Health Organization (WHO), in 2016, approximately 1.34 million deaths were reported globally due to Hepatitis virus induced hepatic disorders [2]. HCV displays sequence variability leading to 7 different genotypes and an enormous list of subtypes [3]. Acute Hepatitis C infections are most often asymptomatic and 15–25% of the time are spontaneously resolved by the immune system [4]. However, most infections (75–85%) lead to chronic HCV, which in 15–25% of patients develops into liver cirrhosis, and in 1–5% and 2–5% into hepatocellular carcinoma (HCC) and end stage liver disease (ESLD), respectively [5,6]. However, unlike many chronic infections HCV can be cured. Until 2015, HCV treatments included a combination of pegylated interferons (pegIFN) alpha and Ribavirin (RBV) and induced a sustained virological response (SVR); the absence of detectable HCV RNA on blood testing 6 months after the completion of antiviral therapy, in 50–80% of patients depending on the virus genotype [7,8]. However, in addition to its poor efficacy, this therapy was expensive and induced severe adverse effects, including flu-like symptoms, hemolytic anemia, and psychiatric disturbances [9,10]. In 2015, the approval of several combinations of direct-acting antivirals (DAA) revolutionized HCV treatment by providing patients with oral therapy that was 95% effective at inducing SVR after 12–14 weeks of treatment with minimal adverse effect [11,12,13,14,15]. DAA therapy is being used to eliminate HCV in certain populations and regions, but an effective vaccine will be needed to control HCV on a global scale [15].

HCV is an enveloped virus with a positive sense RNA genome of ~9.6 kb in length. HCV infections start with virion entry, which is facilitated by attachment of the viral particles to several cell surface receptors and co-receptors followed by endocytosis [16]. The genome encodes a single polyprotein flanked by 5′ and 3′ untranslated regions (UTRs). The 5′UTR contains an Internal Ribosome Entry Sequence (IRES) that regulates translation of the viral polyprotein, a 3000 amino acid polypeptide that is proteolytically processed by host and viral proteases into 10 individual viral proteins; seven nonstructural proteins (p7, N2, NS3, NS4A, NS4B, NS5A, and NS5B) that facilitate polyprotein processing, viral RNA replication, and viral particle assembly, and three structural proteins (core, E1, and E2) that make up the virion particle [16]. The RNA genome replicates within replication complexes formed in association with infection-modified intracellular membranes via synthesis of a negative strand RNA genome intermediate [17]. A unique character of the HCV life-cycle is its reliance on a liver-specific microRNA, miR-122. miR-122 anneals to two sites on the 5′UTR upstream of the IRES and is required for viral RNA accumulation in infected cells [18]. The mechanism by which miR-122 promotes HCV propagation is not fully understood, but counter to the conventional role of microRNAs, which suppress translation and promote mRNA degradation, miR-122 promotes translation and stabilizes the viral genomic RNA and has also been reported to alter the viral RNA structures. It is speculated that HCV evolved to be dependent on miR-122 to limit virus replication to the liver [19], and miR-122 may also affect HCV pathogenesis with the viral genome acting as a miR-122 sponge affecting the normal cellular functions of miR-122 [20]. In this review, we will discuss how miR-122 promotes the HCV life cycle, how the unconventional relationship of miR-122 can be exploited to treat HCV infections, how the virus can escape the need for miR-122, and what we can learn from mutant viruses capable of miR-122-independent HCV replication.

## 2. HCV and miR-122

MicroRNAs (miRNA) are small non-coding RNAs of approximately 20–23 nucleotides that mediate post-transcriptional gene silencing by promoting mRNA degradation and repressing translation [21,22]. miR-122 is a liver-specific microRNA abundantly expressed in hepatocytes [approximately 660,000 copies per cell] which accounts for almost 72% of total miRNA pool in the liver, making it one of the most highly expressed miRNAs in any tissue [23]. The primary miR-122 (Pri-miR-122) is transcribed by RNA polymerase II from a single genomic locus on chromosome 18 [24] and like other miRNAs are processed by Drosha and Dicer into a mature miRNA that targets cellular mRNAs for translation suppression and degradation. The downstream functions of miR-122 are diverse and includes regulation of liver development, lipid and cholesterol metabolism, iron metabolism, and circadian rhythms [24,25,26,27,28,29]. miR-122 expression is typically observed to be lost in hepatocellular carcinoma (HCC), and miR-122 knockout mice developed persistent hepatosteatosis, fibrosis, and hepatocellular carcinoma (HCC), suggesting that miR-122 also has an important role as a tumor suppressor [30,31]. The liver specific expression pattern and sequence of mature miR-122 is highly conserved across the vertebrate lineage, suggesting a co-evolution of this microRNA with the emergence of the liver [32].

### 2.1. Annealing of miR-122

**Figure 1:**
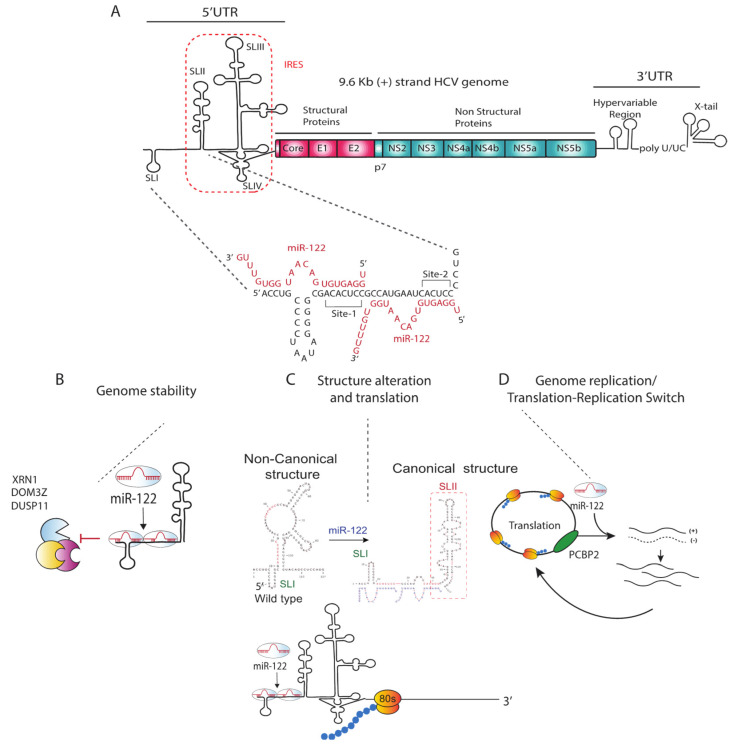
The miR-122 annealing pattern and hypothetical mechanistic models of miR-122 promotion of HCV propagation. (**A**) HCV positive strand genomic RNA with miR-122 annealing pattern. miR-122 binds to seed and auxiliary binding sites on both binding site 1 (S1) and site 2 (S2). (**B**) miR-122 protects RNA genome against RNA degradation machinery. (**C**) miR-122 binding alters 5′UTR structure to a translation favourable structure (**D**) miR-122 acts as a switch between viral replication and translation.

Unlike the canonical suppressive role of miRNAs, miR-122 promotes HCV replication and is essential for detectible HCV replication in cell culture [18,33,34,35,36,37]. miR-122 anneals directly to two homologous sites near the 5′ terminus of the HCV genome (Figure 1) [18,34,38,39]. The first miR-122 site (S1) includes a seed annealing site located adjacent to stem-loop I and auxiliary binding nucleotides at the extreme 5′ end of the viral genome such that miR-122 annealing generates a 3′ overhang on the viral genome 5′ terminus (Figure 1). The second miR-122 seed binding site (S2) is located at the base of stem-loop II and includes auxiliary binding near the S1 (Figure 1). Both seed and auxiliary annealing at both sites is required for efficient HCV replication [18,34,35]. Studies by Mortimier and Doudna, and Pang et al. suggested that miR-122 binds to site 2 with a higher affinity than site 1 [40,41], but there are contradicting reports of the importance of each binding site for replication promotion, with some studies suggested that site 1 binding is more important [33,42], and another study by Thibault et al. suggests an equal contribution of each site on viral replication [43]. In addition, a recent study showed that the specific binding pattern exhibited by miR-122 is not required for the mechanism by which miR-122 promotes replication since annealing of a single small RNA to nucleotides 23–35 promoted HCV replication as efficiently as annealing of two copies of miR-122 [44]. Since nucleotides 23–35 are also bound by miR-122 when annealed to both S1 and S2, this suggests that annealing to this region may be central to the mechanism of viral propagation by miR-122. 

In addition to the two seed binding sites on the 5′UTR, in silico analyses have suggested multiple alternative miR-122 binding sites on the viral genome, four in the NS5B coding region, and three (one very highly conserved) in the 3′UTR [44,45,46,47]. However, neither annealing of alternative small RNAs to these sites nor mutational analysis showed an impact on virus propagation, so the relevance of miR-122 annealing to these sites is still unknown [44,45].

### 2.2. miR-122-Protein Complexes and HCV Propagation

In addition to miR-122, HCV propagation is also dependent on host cellular proteins involved in miRNA biogenesis and translation suppression. Biogenesis of miRNAs is mediated by host proteins that include Drosha, which processes long pri-mRNAs into pre-miRNA hairpins, and Dicer that process the hairpins into mature miRNAs; so, unsurprisingly, Drosha and Dicer are also required for HCV replication, presumably to provide a supply of miR-122. In addition, several host proteins involved in miRNA translation suppression activity, including proteins that comprise the RNA Induced Silencing Complex, (RISC), such as Argonautes (Ago1, 2, 3, and 4), GW182, DiGeorge syndrome critical region 8 (DGCR8), and TAR RNA binding protein (TRBP) have also been shown to be involved in HCV replication promotion by miR-122 [36,37,48,49], suggesting a RISC-like protein complex is required for delivery of the miR-122 and perhaps the mechanism of replication promotion. The role of Ago2 has been studied in more detail and the Ago2:miR-122 complex binds to two sites (S1 and S2) on viral 5′UTR and is required for miR-122 promotion of virus replication [37,50,51,52]. Cells having a knockout of the Ago2 gene still supported viral replication, although to a lower level compared to the wild type cells, suggesting that other Ago isoforms (Ago 1, 3, and 4) are also capable of mediating miR-122-induced HCV propagation [51]. It has also been proposed by several groups that the Ago:miR-122 complex and not miR-122 alone likely promotes HCV propagation [50,51,53,54]. 

### 2.3. Other microRNAs Promoting HCV Replication

Recent findings by the Matsuura group suggest that other cellular miRNAs can bind to the viral 5′UTR and support viral replication in a miR-122-like and a non-miR-122-like manner [55]. The miRNAs identified to bind to viral 5′UTR at both miR-122 binding locations are miR-504-3p, miR-574-5p, and miR-1236-5p, and replication promotion requires at least six complementary nucleotides annealing with the viral RNA. miR-25-5p and miR-4730 were identified to bind to 7–8 nucleotides in a single site that bridge between sites 1 and 2 and promote viral replication. Altogether, these findings suggest that HCV can also usurp miRNAs other than miR-122 which could allow HCV to replicate in tissues other than the liver. Finally, at least four other miRNAs (miR-199a*, miR-196, let-7b, and miR-448) have been confirmed to bind to HCV genome, but these do not promote viral propagation [56]. These microRNAs function in a canonical manner to suppress viral translation, and let-7b can lead to cleavage and degradation of the viral genome [57]. Although these microRNAs are reported to inhibit viral propagation, their net negative effect appears to be superseded by promotion by miR-122 since knockdown of the microRNA pathway proteins leads to an overall reduction in viral propagation [19,37,50,52,56,58]

## 3. Mechanism of miR-122 Promotion of HCV Replication

Numerous studies have aimed to understand the mechanism by which miR-122 promotes virus replication and 3 primary mechanisms are proposed: (1) Protection of the viral genome from degradation, (2) Altering the structure of the 5′UTR to activate viral IRES translation, and (3) A direct role in virus replication (Figure 1). 

### 3.1. miR-122 Protection of the Viral Genome from Degradation

The presence of a 7-methylguanylate cap at the 5′ end of eukaryotic mRNAs promotes translation, protects the 5′ end from cellular exonuclease degradation, and masks viral genomic RNA detection by cellular innate immune sensors. Viral RNAs obtain caps by various methods, including viral encoded capping enzymes (e.g., flavivirus and coronavirus) or by usurping caps from cellular mRNA (e.g., Influenza virus). However, the HCV genome, which is the template for both translation and replication, has an uncapped 5′ triphosphate end [50]. It was speculated that miR-122 annealing at the extreme 5′ end of the viral genome might act as an artificial cap to protect the triphosphate 5′ end of viral RNA from the cellular RNA degradation machinery or from detection by innate immune sensors [35,40,43,51,59,60,61,62]. While there is no evidence thus far that miR-122 protects against recognition by the innate immune sensors [62], studies by Shimakami et al. reported that transfection of miR-122 slowed the decay of a replication-defective viral RNAs, suggesting a role in protecting the viral genome from degradation [42,50]. Additionally, knockdown of exonucleases Xrn1 and Xrn2, which degrade RNA in a 5′ to 3′ direction, partially rescued HCV replication and further supported that miR-122 protects the viral genome from Xrn1 and, to a lesser extent, Xrn2 mediated degradation [43,60,61]. In a recent study, it was also observed that miR-122 protects the genome from cellular pyrophosphatases DOM3Z and DUSP11 [62,63]. DOM3Z is a cellular decapping and exoribonuclease protein with pyrophosphohydrolase and 5′-3′ exonuclease activity required for decapping and degradation of mRNA [64], and DUSP11 is another cellular 5’ pyrophosphatase required for regulating the cellular level of RNA transcripts from RNA pol I and III by hydrolyzing the di-and tri-phosphate from the 5′ end of the RNA [63]. DUSP11 has also been reported to directly act on the 5′ end of HCV RNA rendering it susceptible to Xrn1-mediated degradation [63]. Further, depletion of all three enzymes, Xrn1, DOM3Z, and DUSP11, rescued HCV replication in the absence of miR-122 [62], however, knockdown of Xrn1, DOM3Z, and DUSP11 did not reinstate HCV replication to an miR-122 dependent level and suggests that miR-122 mediated protection of viral RNA is not the only mechanism by which miR-122 promotes viral propagation [62,65]. 

### 3.2. Viral Translation Stimulation by miR-122-Induced Alteration of Genomic RNA and IRES Structure

MiR-122 annealing has been reported by several groups to stimulate HCV translation [33,37,52,53,58,66,67]. However, because of the difference between the magnitude of miR-122 induced viral translation (2 to 3 fold) and miR-122 enhancement of viral replication (1000 fold), the relevance of the impact of miR-122 induced viral translation on promotion of viral replication has been questioned. However, recent evidence suggests that stimulation of translation is a primary mechanism by which miR-122 promotes the HCV life-cycle. In a study by Kunden et al., small RNAs annealed to different locations on the 5′UTR were found to promote the HCV life-cycle with various efficiencies, and the efficiency of replication promotion having similar trends as their ability to stimulate transition suggesting the two functions are linked. In addition, miR-122 annealing has been hypothesized to alter the secondary structure of the 5′UTR and promote the formation of the thermodynamically stable canonical IRES structure [51,53,54]. In silico structure prediction and SHAPE analysis of 1–117 nt of viral 5′UTR in the absence of miR-122 predicts that it preferentially forms a non-canonical structure termed SLII^alt^ that would not support HCV IRES translation, and that annealing of miR-122 shifts the folding equilibrium toward formation of SLII, an RNA structure essential for IRES mediated translation [51,53,54,68]. Biophysical analyses of HCV IRES RNA structure and function indicated that SLII is essential for HCV IRES activation by inducing a conformational change in 40S ribosome and an interaction with the HCV IRES SLIV domain that facilitates the formation of the final 80S ribosomal complex that initiates translation, however, these biophysical analyses were done using HCV RNAs that lacked the 5′ region to which miR-122 binds, thus detailed biophysical analyses of the impact of miR-122 on the IRES structure remain to be confirmed. (Figure 1).

A recent report proposed a dynamic model for the mechanism of Ago:miR-122 promotion of HCV that involved sequential annealing to the two sites on the 5′UTR. The Ago:miR-122 complex was modeled to first bind to site 2, and it serves as an RNA chaperone to re-fold the RNA into the functional SLII conformation of the active IRES, which is then followed by the subsequent binding of a second Ago:miR-122 complex to site 1, promoting viral genome protection by cellular endonucleases and pyrophosphatases. Binding of Ago2 at site 1 was also modeled to stabilize the complex through interactions between Ago and SLII and then release the Ago:miR-122 auxiliary interactions at Site 2 [68] (Figure 1). However, a recent study by Kunden et al. showed that annealing of a single Ago2:small RNA complex at nucleotide position 27–45, a region that spans the annealing site 1 seed and the site 2 auxiliary, both stabilize the genome and promote virus replication to levels equivalent or more than that of miR-122. That a single small RNA:Ago complex can promote genome stabilization, virus translation, and the virus life cycle suggests that the complex annealing pattern, 5′ end annealing, and the dynamics of Ago2:miR-122 binding is not essential for the mechanism of miR-122 promotion of HCV [44].

### 3.3. A Direct Role for miR-122 in Promoting Genome Amplification 

It has been hypothesized for many years that miR-122 has a direct role in HCV genome replication or in regulating the transition from genome translation to genome replication. A study by Masaki et al. showed that miR-122 induced an increase in viral replication that preceded an increase in viral protein synthesis, suggesting a direct role for miR-122 in promoting viral genomic RNA replication that did not rely on increased viral protein synthesis [69]. The study also found that miR-122-stimulated viral replication ceased following siRNA depletion of host poly[rC] binding protein 2 [PCBP2]. PCBP2 interacts with the HCV 5′UTR to promote genome circularization and genome replication [70], and Masaki et al. also showed that miR-122 displaced PCBP2 from HCV RNA in cell-free pull-down experiments and thus speculated that miR-122 binding might enhance the replicating pool of viral RNA by displacing PCBP2 [69]. Additionally, consistent with this notion was reduced polysome association of HCV RNA upon miR-122 transfection, suggesting a role as a molecular switch, activating replication of viral genomes [69,71]. Alternatively, miR-122 binding to the HCV RNA may promote genome synthesis and activate replication by displacing the positive strand from the newly synthesized negative strand replication intermediate to increase accessibility of the 3′ end for initiation of positive strand genomes. Thus, miR-122 is hypothesized to play a role at early stages of the virus life cycle, through enhancement in viral translation and genome stability but may also have a direct role in inducing genome replication. 

## 4. Clinical Significance of miR-122 in HCV Infections

### 4.1. miR-122, HCV Liver Tropism and Pathogenesis

HCV has evolved to infect the liver and is a major factor affecting HCV′s liver-specificality. That HCV usurps miR-122 may also contribute to liver induced pathogenesis by acting as a miR-122 sponge and de-repressing cellular mRNAs targeted by miR-122 [20]. Genome-wide high-throughput sequencing of Ago2 crosslinked and immunoprecipitation (HITS-CLIP) analysis in HCV infected and naïve cells revealed a significant reduction in global AGO binding of miR-122 targeted mRNA and de-repression of miR-122-targeted mRNAs. Further, microarray data from liver biopsies of HCV-infected and naive patients showed a significant de-repression of similar miR-122 target mRNAs and confirmed the HCV sponge effect in vivo. The sponge effect by the viral RNA on miR-122 affected cell proliferation and survival, collagen production, and hepatic stellate cell activation, which could influence liver inflammation [20]. Since miR-122 is a known tumor suppressor, miR-122 sequestration during an HCV infection may also contribute to HCC development in chronic infection [30,31,72,73]. 

### 4.2. miR-122 as a Biomarker for Chronic Hepatitis C Infection and Liver Diseases

Circulating microRNAs in human peripheral blood have been increasingly regarded as potential indicators of a variety of physiological and pathological conditions, including liver injury induced by hepatotoxic agents and viral hepatitis [74,75,76]. Circulating microRNAs are secreted in different body fluids through exosomes and macrovesicles, making them potential and relatively non-invasive biomarkers for the detection of different stages of a disease progression [77]. Circulating miR-122 has been studied as promising circulating biomarkers for liver disease conditions because its expression is dysregulated in liver diseases. Since microRNAs are stable in human plasma/serum and miR-122 being a tissue-specific miRNA, its abnormal presence in the serum can indicate liver injuries [20,30,75,78,79]. Assessing circulating miR-122 levels has shown promise in diagnosing liver pathogenesis in various diseases such HBV, HIV, and/or HCV-associated chronic viral hepatitis, and as a biomarker for chronic liver infection [75,80,81]. 

## 5. Other Viruses That Rely on miR-122 or Other miRNAs

### 5.1. Non-Primate Hepacivirus and miR-122: Similarities, Differences, and Evolution

In addition to HCV, genomes of other viruses in the genus hepacivirus likely anneal to miR-122. Recent studies have identified several novel hepaciviruses including equine hepacivirus, or non-primate hepacivirus (NPHV), Norway rat hepacivirus (NrHV) or rodent hepacivirus-nr-1 (RHV-nr-1), bat hepaciviruses; bovine hepaciviruses (BovHepV), and Guerza hepacivirus, and they all exhibit liver tropism and have at least one miR-122 binding site on their genome [74,82,83,84,85,86,87,88]. Studies have confirmed miR-122 stimulation of translation in NPHV and BovHepV, and miR-122 sequestration inhibits NPHV accumulation in cell culture [74,89]. GB virus B (GBV-B), a relative of HCV isolated from laboratory tamarins, also has two miR-122 binding sites on its 5′UTR RNA and is dependent on miR-122 and Ago2 abundance for viral propagation [90]. The presence of the miR-122 binding site in hepaciviruses of different species suggests evolution from a common liver-tropic ancestor [19] and a conserved mechanism of action. Altogether, it can be proposed that throughout host–virus coevolution, hepaciviruses may have used miR-122 as a common strategy to establish liver tropism.

### 5.2. Let-7 and MiR-17 Promotion of Bovine Viral Diarrhea Virus 

The dependency of viruses on miRNAs is rare but is shared by one other virus from the *Flaviviridae* family. A study by Scheel et al. reported that Bovine viral diarrhea virus (BVDV) an animal pathogen from pestivirus genus, also requires microRNAs let-7 and miR-17 for their propagation [91,92]. However, unlike miR-122 and HCV, these microRNAs bind to the 3′UTR of the BVDV genome to promote translation and replication. The discovery of other viruses dependent on host microRNA provides another example of the unusual relationship between the host and virus, which might provide insight into common mechanisms of action and a broader picture of host-pathogen co-evolution. 

## 6. miR-122 Based HCV Antiviral Therapy, Resistance Associated Mutations and miR-122-Independent Replication

As an HCV host dependency factor, miR-122 is a promising therapeutic target for HCV treatments. Two antisense locked nucleic acid (LNA) inhibitors of miR-122, Miravirsen™ (Santaris Pharma a/s) and RG-101 (Regulus Therapeutics), have been used successfully to limit HCV replication in cell culture and in chronic HCV-infected patients [93,94,95]. Both anti-miR-122 LNA drugs have completed Phase II and Ib clinical trials, respectively, and showed a sustained reduction in HCV viral load in a dose dependent manner. Neither treatment showed significant adverse effects or safety issues; however, both the studies showed a virologic rebound several weeks post treatment. Miravirsen significantly reduces viral loads in patients and can be used in combination with other standard HCV treatment to facilitate a better and efficient anti HCV treatment regimen or to treat the patients who do not respond to DAA [96,97]. However, since miR-122 is a tumor suppressor, the long-term use of miR-122 antagonists should be done with caution. 

**Figure 2:**
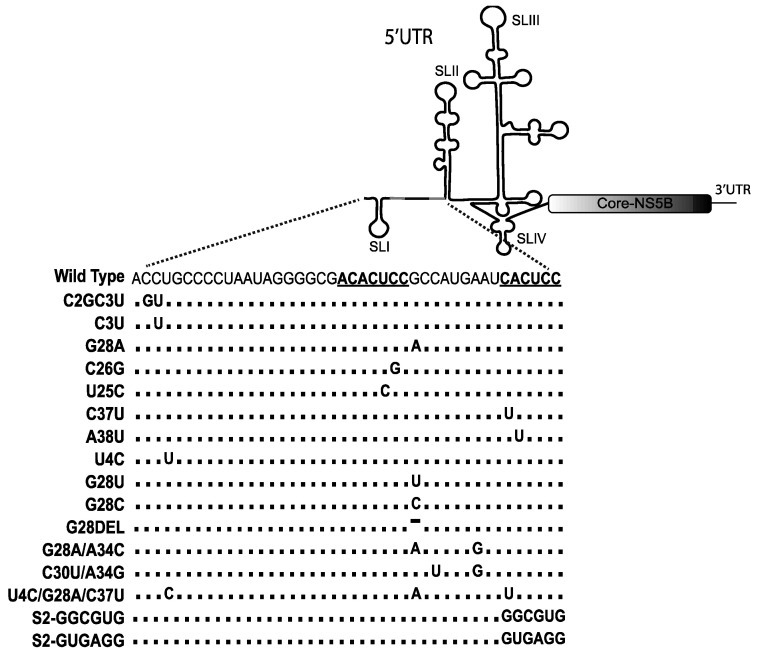
5′UTR mutations in full-length HCV variant genomes that can replicate in the absence of miR-122.

Sequence analysis of viral RNA isolated from miR-122-inhibitor treated patients suggested evolution of resistance-associated substitutions (RAS) on the viral 5′UTR; specifically, C2GC3U, C3U [cytosine substitution by guanosine and uracil at nucleotide 2 and 3 positions], and G28A (Guanosine substitution by Adenine at nucleotide position 28) mutations (Figure 2) [93,94,98,99]. The C2G, C3U, and C3U mutations are in the auxiliary annealing region of miR-122 binding site 1, and G28A is located adjacent to the auxiliary annealing site of miR-122 binding site 2. Thus, it appeared that HCV may be able to evolve to escape miR-122 antagonists, perhaps by replicating independently of miR-122; but, interestingly, viruses carrying the C3U mutation appeared to be transiently expressed only during treatment with anti-miR-122 and appeared to have reduced replication efficiency than wild type in the presence of miR-122 [100]. This is consistent with several reports of detectible but inefficient miR-122-independent HCV replication in cell culture. 

### 6.1. Genetic Models of miR-122-Independent Replication

In cell culture, miR-122 is essential for detectible replication of wild type HCV genomes. However, in addition to miravirsen resistance associated mutations, several virus mutants have been reported to replicate, mostly with poor efficiency, in the absence of miR-122. The first report of miR-122-independent replication was of a viral genome that contains a fragment of U3 small nucleolar RNA (U3 snoRNA) on the 5′UTR in place of SLI and miR-122 binding site 1 [96] that was selected by passaging of SLI deleted viral genome in Huh 7.5 cells. Based on our current knowledge, it seems likely that the added sequences mask the 5′UTR from degradation and may modulate 5′UTR RNA structures, but this remains to be confirmed. In addition, HCV subgenomic replicon RNAs were found to exhibit low levels of miR-122-independent replication [101]. The replicons were bicistronic and contain an EMCV IRES that drives the translation of viral non-structural proteins and the altered translation regulation by the added IRES allowed both full length and subgenomic RNAs to replicate in the absence of miR-122 [102]. Thus, additional 5′UTR sequences or altered translation regulation allowed for miR-122-independent HCV RNA replication. 

In addition, full-length viral genomes having mutations in the 5′UTR miR-122-binding region also displayed miR-122-independent replication (Figure 2) [51,71,103,104]. Genomes having a G28A (Adenine substitution of Guanine at nucleotide position 28) mutation were identified in numerous studies and are widely used as a model system to study HCV replication in the absence of miR-122 [51,71,103,104]. The G28A variant is present in naturally occurring sequences and provides resistance toward miR-122 knockdown [103]. However, the mutation does not provide any advantages or disadvantage over wild type virus when miR-122 is present [71]. The G28A mutation was also identified in HCV infected cell cultures and patient-derived PBMCs [71,103], and it remains the only naturally occurring virus mutant that can replicate in the absence miR-122. 

In addition to G28A, a number of other full-length HCV variants with 5′UTR mutations have been reported to replicate in the absence of miR-122 in cell culture (Figure 2) [51,71,104]. These mutants were generated by serially passaging HCV in miR-122 knockout Huh 7.5 cells expressing a mismatched miRNA [104] or through siRNA-knockdown mediated mutagenesis of the miR-122 binding region [51]. HCV mutations reported to provide an advantage to viral replication in the absence of miR-122 were identified in both miR-122 seed binding sites 1 and site 2 (C26G, U25C mutants, C37U, and A38U), miR-122 auxiliary binding site 1 [U4C], the nucleotide sequence between site 1 and site 2 (G28 mutants, G28U, G28C, G28DEL, G28A/A34G, C30U/A34G), and a combination of mutations present in Auxiliary site1, seed site 1, and seed site 2 (U4C/G28A/C37U) [51,65,71,104]. Another full-length HCV mutant reported to replicate independently from miR-122 was HCV-S2-GGCGUG, selected from a population of viral variants having all possible sequences at S2 [19], and in another report complete mutation of miR-122 binding site 2 to GUGAGG (HCV-S2C- GUGAGG) can also allow the virus to replicate independently of miR-122 [65]. Thus, several different mutations spread over the first 42 nucleotides of the viral genome appear to compensate for the lack of miR-122. However, even though these mutants can replicate in the absence of miR-122, the efficiency of miR-122-independent replication is generally low, and they are still responsive to miR-122 and replicate efficiently when it is present. Finally, none of these mutations, other than G28A and the miravirsen resistance associated mutations, have been detected in patient virus samples and this region remains highly conserved, suggesting evolutionary pressure for HCV to retain miR-122 dependence [71,98,99,100]. 

### 6.2. MiR-122-Independent Replication and Extrahepatic Manifestation of HCV

However, and controversially, the ability of HCV to escape the need for miR-122 in tissue culture and potentially in patients treated with miR-122 antagonists suggests the possibility of miR-122-independent HCV replication in extrahepatic tissues. Although HCV primarily infects the liver, HCV associated non-hepatic pathogenicity has been reported [105]. The broad clinical spectrum of extrahepatic complications and diseases associated with chronic HCV infection include mixed cryoglobulinemia, non-Hodgkin’s lymphoma, cutaneous vasculitis, glomerulonephritis, neuropathy, and lymphoproliferative disorders, to name a few [105,106,107,108,109]. Extrahepatic manifestations are common in HCV infected patients with 74% of patients reporting at least one [107]. The underlying pathogenic mechanism of extrahepatic complications is thought to be immune-mediated. However, a growing and controversial body of evidence also suggests a direct impact of HCV replication. HCV has been published to replicate in peripheral blood mononuclear cells (PBMCs), including B and T cells, monocytes/macrophages and dendritic cells, and the central nervous system (CNS) [110,111,112,113,114]. Detection of the negative viral genomic RNA in different tissues and PBMCs suggested that the virus is capable of extrahepatic replication [105] and PBMCs isolated from patients with chronic HCV, occult HCV, and chronic HCV/HIV coinfection has been reported to support HCV replication [105]. However, how viral replication was supported in these cells which do not express miR-122 is unknown. One of the possible explanations could be exosome mediate transportation of viral RNA and miR-122 through blood circulation. A study from Bukong et al. revealed that exosomes from HCV infected hepatocytes contain replication-competent HCV RNA in complex with miR-122-Ago2-HSP90, suggesting exosome-mediated infection of cells [115]. Furthermore, genome analysis revealed that selection of viral variants that grow independently of miR-122 and affect tropism can influence HCV replication in extrahepatic tissue. Recent studies posit that adenine substitution at the 28 nucleotide position (28A) of the HCV genome, a mutation that enhanced miR-122-independent replication, can allow the virus to replicate in PBMCs as this viral variant has been isolated from patient-derived PBMCs by different groups [71,103]. However, even if virus infection of PBMCs is confirmed, the link with pathogenesis and extrahepatic manifestation of HCV is still unclear. 

## 7. Mechanism of miR-122-Independent Replication

### 7.1. Replication Promotion by Other microRNAs

Cell culture models of miR-122-independent HCV replication have been used to study mechanisms by which the virus escapes the need for miR-122. One possibility is that HCV variants reported to replicate in the absence of miR-122 use other cellular miRNAs, such as those identified by Ono et al. to anneal to the 5′UTR [55]. However, several studies on miR-122-independent replication also showed virus replication in Dicer and Drosha knockout cells, which are devoid of miRNAs that rely on the canonical miR-122 biogenesis pathways. HCV-S2-GGCGUG which has been reported previously for replicating in the absence of miR-122 was capable of replicating in Dicer KO Huh 7.5 cells, and several mutants were shown to replicate in Drosha knockout cells [19,65]. Additionally, another study showed that Ago2 interaction with HCV genome was not detected for miR-122-independent replication of G28A, suggesting that miR-122 independent replication of HCV is not because of the binding of other microRNAs to the 5′UTR of the viral RNA and no other microRNA compensate for miR-122-independent replication of G28A HCV [71]. However, these studies do not remove the possibility of the involvement of small cellular RNAs that do not rely on Dicer or Drosha or other Ago isoforms. 

### 7.2. 5′UTR Mutations Stabilize the Viral Genome

**Figure 3:**
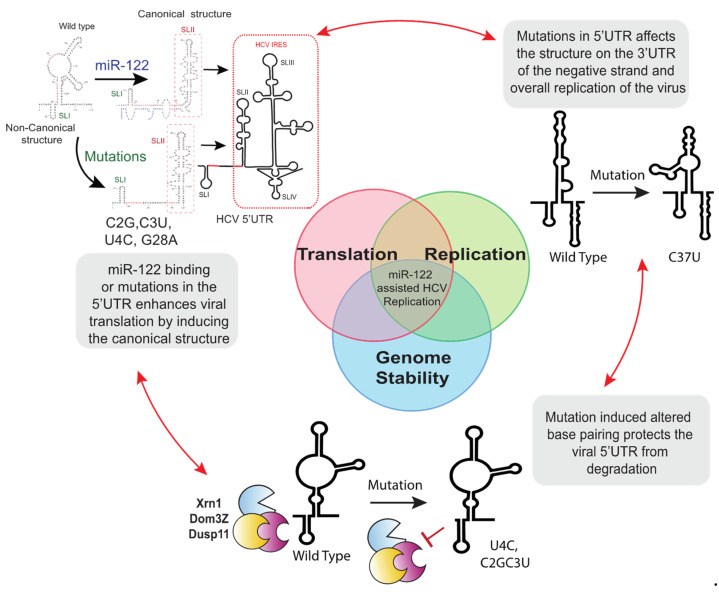
A model for the mechanism of miR-122 independent replication. miR-122-independent replication is an interplay between genome translation, replication, and stability. See text for details.

A method by which miR-122 is reported to promote HCV replication is through stabilization of the viral genome (Figure 3). Based on this, Chahal et al. suggested that mutations to the auxiliary region of miR-122 binding site 1 (C2G, C3U, U4C) located near the genomic 5′ terminus protect the genome from degradation, and this notion was supported by in vitro stability assays showing that the mutant RNAs were resistant to degradation by Xrn1 [68]. However, small RNAs that anneal to nucleotides 19–37 and not to the 5′ terminus also stabilized the genome and promoted HCV replication, indicating that miR-122 5′ end annealing is not essential for genome stabilization [44].

### 7.3. 5′UTR Mutations Modulate the 5′UTR RNA Structure and Stimulate Virus Translation

Another proposed mechanism of action of miR-122 is as a chaperon that alters viral 5′UTR RNA structures, refolding SLII into the canonical structure of an active viral IRES (Figure 3) [51,53,54]. Computational and SHAPE analysis of the 5′UTR viral RNA of mutants replicating independently of miR-122 predicts that their 5′UTRs have a higher propensity to form a structure similar to the canonical SLII structure formed by wild type virus after miR-122 annealing. Thus, it was proposed that 5′ RNA structure may be dynamic and that mutations that allow for miR-122-independent replication shift the folding equilibrium toward that of the active IRES even in the absence of miR-122 [51]. In support of this, there was a correlation between the mutation induced enhancement of translation and the efficiency of miR-122-independent replication for the HCV mutants capable of miR-122-independent replication [53,65]. Further, knocking down of RNA degrading enzymes rescued replication of several mutants, some to miR-122-dependent levels, suggesting that the roles of miR-122 can be compensated by enhancing virus translation and stabilizing the viral genome. Experiments also demonstrated that enhanced translation rescued viral replication by approximately 100-fold, whereas enhanced stability rescued viral replication approximately 10-fold, and suggested that the major role for miR-122 is translation stimulation with stabilization being important but less potent [65]. 

### 7.4. Removal of a Negative Regulator of Viral Propagation

**Figure 4:**
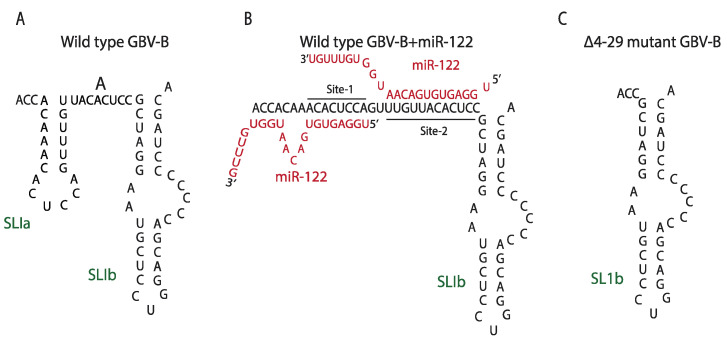
Predicted structures of 5′UTR of HCV and GBV-B with or without miR-122. (**A**) Wild type GBV-B without miR-122 (1–62 nt), (**B**) Wild type GBV-B with miR-122 (1–62 nt), (**C**) ∆4-29 mutant GBV-B without miR-122.

It is also possible that the HCV 5′ terminal region functions as a switchable regulatory element whose primary function is to make the virus reliant on miR-122 (Figure 4). This hypothesis is based on miR-122 regulation of a related hapacivirus, GB virus B (GBV-B). GBV-B also requires miR-122 for efficient viral RNA propagation but was found to be capable of replication independently of miR-122 after simply removing the miR-122 binding region [90]. The deletion mutant lacked much of the 5′UTR, including both miR-122 binding sites (∆4-29), and it was first isolated by growing the GBV-B replicons in Hep3B cells, human hepatoma cells that lack miR-122 expression (Figure 4) [90,116]. A similar deletion of the miR-122 binding region in HCV was not tolerated (unpublished data), presumably because the complementary sequence forms RNA structures on the 3′UTR of the negative strange that are essential for genome replication [117]. However, that GBV-B can tolerate removal of the miR-122 binding sites, suggesting this region is dispensable and that its inclusion makes virus replication rely on miR-122, perhaps to regulate virus tropism.

## 8. Dynamics of miR-122-Independent Replication and miR-122’s Impact on Different Stages of the Virus Lifecycle

Models of miR-122-independent replication have allowed researchers to study how the HCV replication cycle differs when miR-122 is present or absent. Our recent publication showed that miR-122-independent replication in cultured cells is manifested as efficient replication in a smaller number of cells than when miR-122 is present; but, it also showed that once virus replication is established the level of virus protein expression in each infected cell is similar whether miR-122 is present or absent [102]. This suggests that that miR-122 is required to establish an infection but is dispensable after an infection has been established. This notion is also supported by a study showing efficient miR-122-independent replication of HCV replicons [71]. In support of miR-122 being dispensable for an ongoing infection, no detectable miR-122 has been observed within the HCV replicase complex in vivo and the addition of miR-122 or miR-122-antisense inhibitors has no effect on HCV RNA synthesis within membrane-bound replicase complexes isolated ex vivo from HCV infected cells and [118]. However, miR-122 supplementation and antagonism showed a small positive influence on ongoing viral replication when measured by luciferase expression. This suggested a minor supportive role in an ongoing infection. This could be a direct role of miR-122 on genome replication as discussed previously, but we also hypothesized that miR-122 anneals to newly synthesized HCV genomes that exit replication complexes to stabilize and promote translation to augment their ability to establish new replication complexes within an infected cell or after division of an infected cell [102]. Together, this study proposed a model in which miR-122 is essential at the initial stage of infection to allow the virus to reach a protein synthesis threshold required to establish replication complexes and an infection and then may use the same mechanism to assist in the formation of new replication complexes. However, how and when miR-122 dissociates from the viral genome in this model is unknown. 

### Model for the Role of miR-122 in the HCV Life-Cycle: A Host Factor That Allows HCV to Overcome Infection Barriers

**Figure 5:**
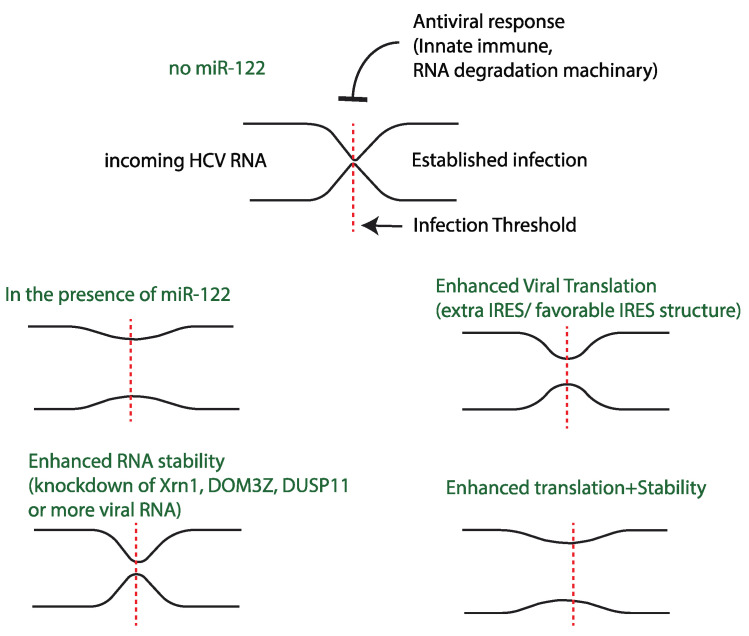
Model for miR-122 promotion of HCV: Schematic representation of infection bottleneck of HCV in the presence of different infection limiting variables.

Establishment of a successful viral infection is determined by the outcome of competition between viral translation/replication kinetics and host antiviral response. Host cells defend against viral infection using strategies that include translation inhibition and viral genome degradation to create a barrier to the initiation of an infection. In turn, viruses have evolved many mechanisms to block the activation of the antiviral pathways to maintain virus translation and genome stability [119,120]. However, a recent publication suggests that the dynamics of virus translation and replication can also overcome the antiviral barrier. For example, to establish a picornavirus infection requires that the virus produce sufficient protein to successfully switch from genome translation to genome replication, but in 15–20% of picornavirus infected cells the incoming viral RNA fails to translate sufficiently to initiate the switch, likely due to antiviral conditions in the cell. However, the genomes that fail to switch to replication will try again, reinitiating translation to further attempt to establish an infection [121]. Similarly, we propose that miR-122 allows HCV to overcome a barrier to infection, specifically RNA degradation by host enzymes and poor translation efficiency. In the absence of miR-122, translation efficiency is poor and the genomic RNA is subject to degradation; this barrier blocks the establishment of an infection. In the presence of miR-122, the RNA is stabilized, translation is efficient, and an infection is established in most cells. However, with mutant HCV genomes having enhanced genome stability or translation efficiency without miR-122, the barrier is overcome in a small number of cells stochastically and an infection is established. Similarly, knockdown of RNA degradation enzymes also reduces the barrier and enhances HCV infection success in the absence of miR-122, in some cases to levels similar to that with miR-122 (Figure 5) [44,53,62,65].

Recent studies have revealed that barriers to infection can also be surmounted by infection of a cell by multiple virions since en bloc transmission of viruses within extracellular vesicles containing multiple viral particles containing multiple viral genomes are more infectious than individual free virions [122,123]. The average number of HCV genomic RNA copies is 1 to 8 per cell in vivo [124]; 100 fold lower RNA levels than tissue culture cells expressing HCV replicons or infectious virus [125]. Since a very low copy number of viral genomes are found in hepatocytes, the presence of miR-122 may be even more important for infection initiation in vivo. Thus, we suggest that miR-122 regulates the dynamics of infection initiation and the establishment of a successful infection.

While our data and model suggest that miR-122 does not have a major role following the initiation of an infection, there is a small positive impact during an ongoing infection. We hypothesize that this is due to the stabilization and translation stimulation of newly synthesized genomes as they initiate new replication complexes within a cell, or following division of an infected cell. In infected patients, chronic HCV infections have been suggested to be maintained by a continuous cycle of viral infection and clearance in a small subpopulation of hepatocytes in the liver [124], and we propose that miR-122 supports the dynamics of this cycle and explains why miR-122 antagonists have a potent effect on chronic infections.

## 9. Conclusions and Future Directions

The liver specific microRNA, miR-122, is essential for efficient HCV replication. It stabilizes the viral genomic RNA and stimulates translation by inducing viral genomic RNA structure modifications; miR-122 limits HCV tropism to the liver where it allows the virus to overcome barriers to infection, and it supports the dynamic infection of hepatocytes during a chronic liver infection. However, gaps in knowledge remain. Biophysical details of the RNA structures formed with and without miR-122 are still lacking, as are the details regarding the viral and host RISC and RNA binding proteins involved. A direct role for miR-122 in regulating the switch between virus translation and replication has been proposed for many years and while there appears an indirect influence on genome replication through enhanced translation and genome stabilization, whether there is a direct impact on the switch to genome replication is still unresolved. Further, details of the impact of miR-122 on the replication and tropism of other hepacivirus that also anneal miR-122 will allow a better understanding of how and why they evolved to be regulated by miR-122, and understanding the mechanisms of HCV genome structure–function will provide insight into the RNA genome structure and function of other viruses.
